# Anti-G-CSF treatment induces protective tumor immunity in mouse colon cancer by promoting NK cell, macrophage and T cell responses

**DOI:** 10.18632/oncotarget.4169

**Published:** 2015-06-04

**Authors:** Katherine T. Morris, Eliseo F. Castillo, Anita L. Ray, Lea L. Weston, Robert A. Nofchissey, Joshua A. Hanson, Von G. Samedi, Irina V. Pinchuk, Laurie G. Hudson, Ellen J. Beswick

**Affiliations:** ^1^ Department of Surgery, University of New Mexico, Albuquerque, New Mexico, USA; ^2^ Department of Molecular Genetics and Microbiology, University of New Mexico, Albuquerque, New Mexico, USA; ^3^ Department of Pathology, University of New Mexico, Albuquerque, New Mexico, USA; ^4^ Department of Internal Medicine, University of Texas Medical Branch, Galveston, Texas, USA; ^5^ Department of Pharmaceutical Sciences, University of New Mexico, Albuquerque, New Mexico, USA

**Keywords:** G-CSF, colorectal cancer, NK cells, macrophages, Th1

## Abstract

Granulocyte colony-stimulating factor (G-CSF) is a cytokine that is highly expressed in human and mouse colorectal cancers (CRC). We previously reported that G-CSF stimulated human CRC cell growth and migration, therefore in this study we sought to examine the therapeutic potential of anti-G-CSF treatment for CRC. G-CSF is known to mobilize neutrophils, however its impact on other immune cells has not been well examined. Here, we investigated the effects of therapeutic anti-G-CSF treatment on CRC growth and anti-tumor immune responses. C57BL/6 mice treated with azoxymethane/dextran sodium sulfate (AOM/DSS) to induce neoplasms were administered anti-G-CSF or isotype control antibodies three times a week for three weeks. Animals treated with anti-G-CSF antibodies had a marked decrease in neoplasm number and size compared to the isotype control group. Colon neutrophil and macrophage frequency were unchanged, but the number of macrophages producing IL-10 were decreased while IL-12 producing macrophages were increased. NK cells were substantially increased in colons of anti-G-CSF treated mice, along with IFNγ producing CD4^+^ and CD8^+^ T cells. These studies are the first to indicate a crucial role for G-CSF inhibition in promoting protective anti-tumor immunity, and suggest that anti-G-CSF treatment is a potential therapeutic approach for CRC.

## INTRODUCTION

Colorectal cancer (CRC) is the third leading cause of cancer related deaths in the United States, suggesting that new treatment approaches are needed [1]. Chronic inflammation, as seen in inflammatory bowel disease, is a key risk factor associated with the development of CRC [2]. A greater understanding of the underlying mechanisms linking inflammation and CRC is likely to lead to improved therapeutics.

G-CSF is a pro-inflammatory cytokine with the well established function of inducing mobilization of neutrophils from bone marrow to the periphery [3]. However, despite the documented presence of this cytokine in head and neck, pancreatic, and ovarian tumors [4–6], little is known about its function in solid tumors or the tumor microenvironment. We found previously that human CRC tumors highly express both G-CSF and G-CSF receptor [7]. We further demonstrated that both carcinoma cells and cancer associated fibroblasts produce high levels of G-CSF. Another group has suggested that monocytes, but not T cells, are a source of G-CSF [8]. Since we found that 88% of human CRC samples examined expressed increased G-CSF and G-CSFR compared to normal tissue, we sought to further examine the function of this cytokine in CRC. In those studies, we demonstrated that G-CSF stimulated proliferation and migration of gastric and colon carcinoma cells, suggesting that G-CSF acts directly on tumor cells. Based on these findings, we were led to consider if blockade of G-CSF could have therapeutic benefits for CRC.

Despite the well-known actions of G-CSF for neutrophil mobilization, the influence of tumor-produced G-CSF on other immune cells is not clear. There is emerging evidence that both neutrophils and macrophages play a role in solid tumor growth, including CRC [9, 10], yet the effects of G-CSF on immune cells in the tumor microenvironment have not been examined. However, there are also reports in other diseases that G-CSF supports accumulation of regulatory T cells (Treg) [11–13], which could promote tumor growth and progression. Furthermore, the impact of G-CSF on anti-tumor immunity has been overlooked thus far.

For this study, we hypothesized that G-CSF supports tumor-promoting immune responses and as such is a potential therapeutic target for CRC. To test this hypothesis, we administered anti-G-CSF or isotype control to AOM/DSS treated mice. Upon completion of the treatment regimen, anti-G-CSF treatment led to an 88% decrease in neoplasm number and an 93% decrease in neoplasm size compared to isotype control. Unexpectedly, colon macrophages and T cell phenotypes were transformed to anti-tumorigenic phenotypes. NK cell and CD8^+^ T cell numbers were markedly enhanced in colons of anti-GCSF treated mice along with evidence of cytolytic activity. This is the first study to indicate the potent tumor immune promoting mechanisms of G-CSF and decreased neoplasm size after G-CSF blockade, suggesting that G-CSF is a potential therapeutic target for CRC. Since the activity of G-CSF has only been examined on neutrophil mobilization in cancer, these previously unknown findings that G-CSF has potent effects on other immune cells is critical for evaluation of new immunotherapies for tumors that produce G-CSF.

## RESULTS

### G-CSF and G-CSFR are increased in mouse colon neoplasms

**Figure 1:**
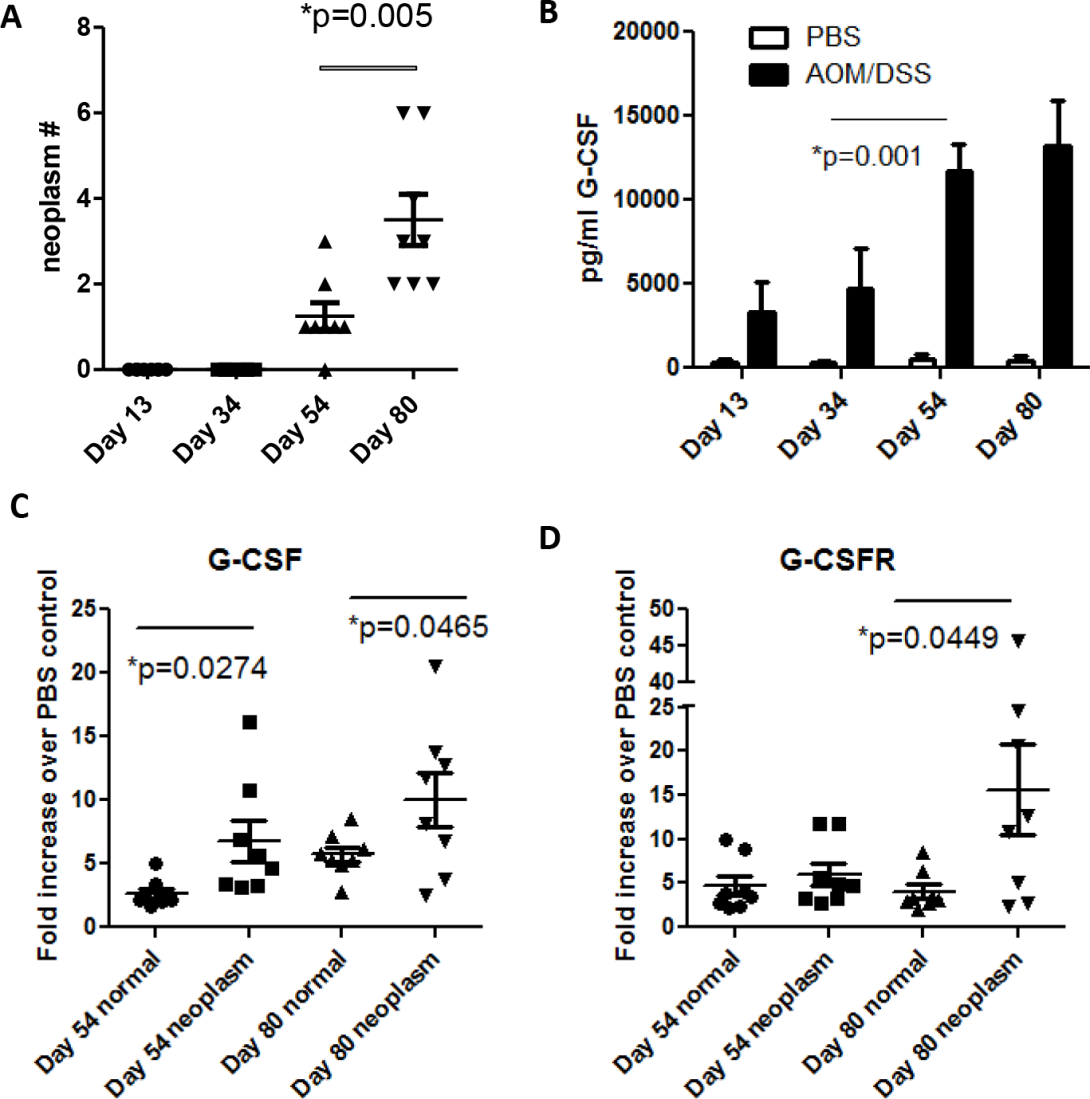
G-CSF and G-CSFR are increased in AOM/DSS treated mice **A.** Neoplasms develop at day 54 in AOM/DSS treated mice and are increased in number by day 80. **B.** G-CSF is increased in colon organ culture supernatant starting at day 13, is substantially increased by day 54, and continues to be produced at day 80 by Luminex bead array. **C.** G-CSF and **D.** G-CSFR gene expression were elevated in colon neoplasms compared to normal colon tissues at days 54 and 80. *N* = 8 from duplicate experiments.

To examine the role of G-CSF in a mouse model of CRC, C57BL/6 mice were administered an AOM injection followed by three rounds of DSS treatments, which is an established model to induce multiple neoplasms between days 40–80 after AOM injection [14, 15]. Here, we examined mouse colons three days after each DSS treatment (days 13, 34, 54, and 80) and found that mice developed neoplasms by Day 54 (Figure 1A). We also investigated G-CSF production in mouse colon organ culture supernatants in order to determine if G-CSF production continued when the inflammatory stimulus (DSS) was removed at each time point. When supernatants were analyzed on Luminex bead assay, we found that G-CSF production was increased starting at day 13 in treated mice compared to control mice, levels peaked at day 54 as inflammation became chronic with multiple treatments, and remained elevated at day 80 (Figure 1B). Since G-CSF protein levels peaked at day 54, we further examined G-CSF and G-CSFR gene expression in mouse neoplasms compared to normal colon tissues. In neoplasms G-CSF was increased by 6.72 and 10.02-fold and G-CSFR by 7.04 and 15.58-fold at days 54 and 80, respectively, compared to normal tissues (Figure 1C and 1D). Both G-CSF and G-CSFR were significantly increased in neoplasms compared to normal tissues from the same mice. Differences between days 54 and day 80 were not significant. These data indicate that the mouse model is representative of the increase in G-CSF and G-CSFR expression that we previously reported in human CRC [7].

### Anti-G-CSF treatment regresses colon neoplasms in mice

**Figure 2:**
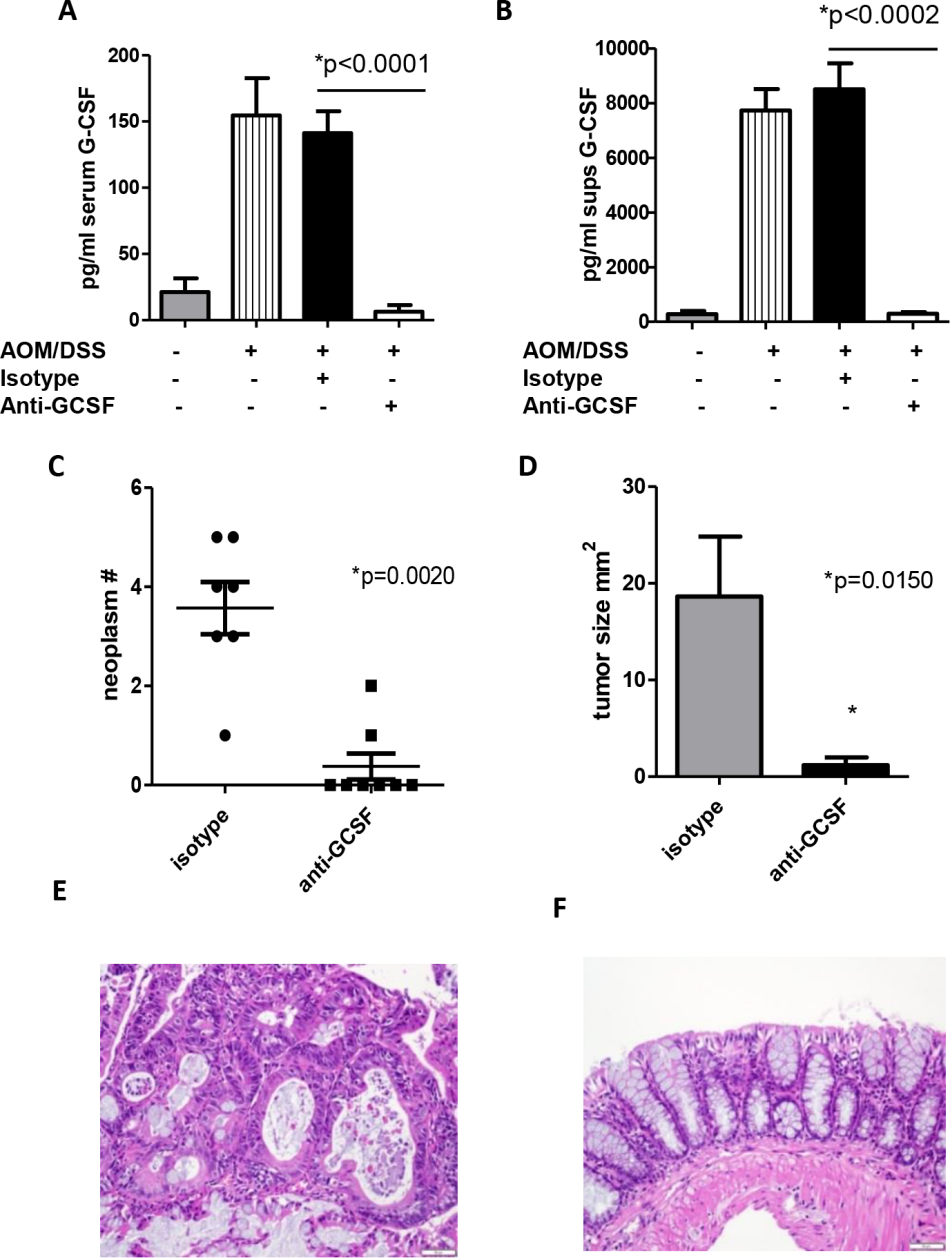
G-CSF plays an important role in neoplasm development in AOM/DSS treated mice Anti-G-CSF administration to AOM/DSS treated mice abrogates G-CSF in **A.** serum and **B.** colon organ culture supernatants by bead array. **C.** Neoplasm number and **D.** size were decreased in mice treated with anti-G-CSF compared to isotype control. H&E staining of colon tissue from an AOM/DSS treated mouse administered **E.** isotype control antibody showing a neoplasm compared to **F.** anti-G-CSF with normal appearing colon. Images are shown at 20x magnification. *N* = 7 for sham PBS control and isotype treated AOM/DSS exposed mice and *N* = 8 for anti-G-CSF AOM/DSS treated mice from duplicate experiments.

The increased G-CSF and G-CSFR expression within the neoplasms in the AOM/DSS model led us to examine the effects of G-CSF blockade therapeutically. At day 54 G-CSF levels peaked and neoplasms were detected (Figure 1), so this time point was selected to test the therapeutic potential of G-CSF blockade. AOM/DSS treated mice were administered isotype control or anti-G-CSF starting at day 54, 3 times a week for 3 weeks and sacrificed on day 80. Treatment with anti-G-CSF abrogated AOM/DSS induced G-CSF in serum (Figure 2A). To examine colon levels of G-CSF, organ culture supernatants were analyzed for G-CSF by bead array, which indicated that G-CSF was also depleted in mouse colons by antibody treatment (Figure 2B). These results indicate that anti-G-CSF treatments were successful both systemically in serum and locally in colon tissues. Next, colon neoplasms were examined and only two of eight anti-G-CSF treated mice had neoplasms, while all seven isotype control treated mice developed multiple neoplasms with a mean of 3.57 per mouse (Figure 2C). Importantly, the two mice treated with anti-G-CSF that developed neoplasms had a much lower frequency (1–2 neoplasms with a mean of 0.38 per mouse) compared to isotype control. The mean size was also much smaller in anti-G-CSF treated mice (0.95 mm^2^) compared to isotype control (9.9 mm^2^) (Figure 2D). Histology of representative colons samples show a colon neoplasm from a mouse administered isotype control antibodies (Figure 2E) compared to tissue from a mouse that was administered anti-G-CSF (Figure 2F). These data strongly indicate a protective role for anti-G-CSF treatment in a mouse model CRC.

### Anti-G-CSF treatment changes macrophage responses in mouse colons

**Figure 3:**
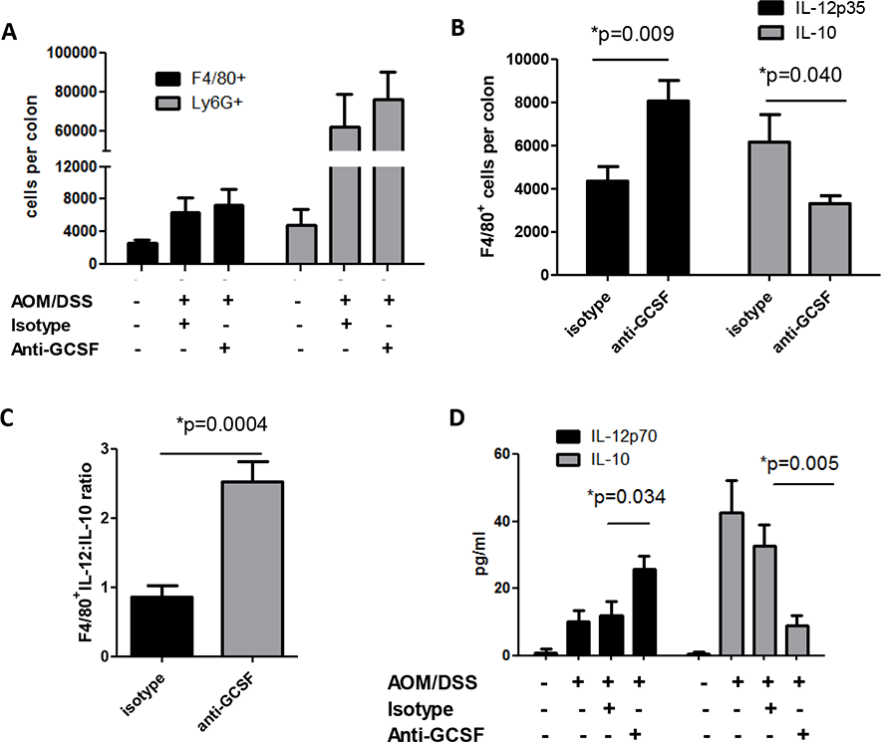
G-CSF neutralizing antibody treatment changes macrophage responses in AOM/DSS treated mice Upon anti-G-CSF treatment **A.** macrophages and neutrophils infiltrating the colons of AOM/DSS treated mice was not significantly changed in number, but **B.** the number of IL-12p35 producing macrophages were significantly increased, while IL-10 producing macrophages were significantly decreased as detected by flow cytometry, **C.** the ratio of IL-12p35:IL-10 producing macrophages was significantly enhanced, **D.** total IL-10 in supernatants from colon tissues was decreased, while total IL-12p70 was increased when measured by multiplex bead array. *N* = 7 for sham PBS control and isotype treated AOM/DSS exposed mice and *N* = 8 for anti-G-CSF AOM/DSS treated mice from duplicate experiments.

Despite well-known functions of G-CSF on neutrophil mobilization, little is known about the effects of G-CSF on other myeloid cells. Colon tissues from mouse groups were examined for neutrophil and macrophage numbers. Since mice develop multiple neoplasms, tissue from both neoplasms and the surrounding microenvironment were utilized for these studies. Colons were processed to prepare a single cell suspension and recovered cells and were stained for flow cytometry. Influx of Ly6G^+^ cells (granulocyte marker indicative of neutrophils) and F4/80^+^ cells (macrophage marker) were found to be increased in AOM/DSS treated mouse colons compared to control mice. Surprisingly, treatment with anti-G-CSF did not affect the influx of neutrophils into mouse colons (Figure 3A). Similarly, the number of macrophages was not significantly affected. However, since macrophages have either tumor-promoting or anti-tumor properties depending on cytokine production, intracellular IL-10 was examined as a pro-tumorigenic cytokine and IL-12 as an anti-tumorigenic cytokine known to be produced by macrophages [16–19]. Mice treated with anti-G-CSF were found to have F4/80^+^ cells expressing approximately double the level of IL-12 (Figure 3B), whereas IL-10 was decreased to approximately one half the levels of isotype control treated mice. When comparing the ratio of IL-12 to IL-10 producing F4/80^+^ cells, a drastically elevated IL-12:IL-10 ratio was observed upon G-CSF blockade (Figure 3C). In organ culture supernatants, a similar overall pattern of decreased IL-10 and increased IL-12 production was also found in anti-G-CSF treated mouse colons by multiplex bead array (Figure 3D). Less is known about the role of neutrophils in tumors than macrophages, but neutrophils have also been suggested to have tumor-promoting capacities [20]. In this study, neutrophil IL-10 and IL-12 did not differ between mouse groups (not shown) indicating that G-CSF may have a different effect on neutrophils than macrophages. These results indicate that G-CSF plays a previously unrecognized role in driving macrophage cytokine responses in the tumor microenvironment.

### Anti-G-CSF treatment induces cytotoxic cell influx and responses in mouse colons

**Figure 4:**
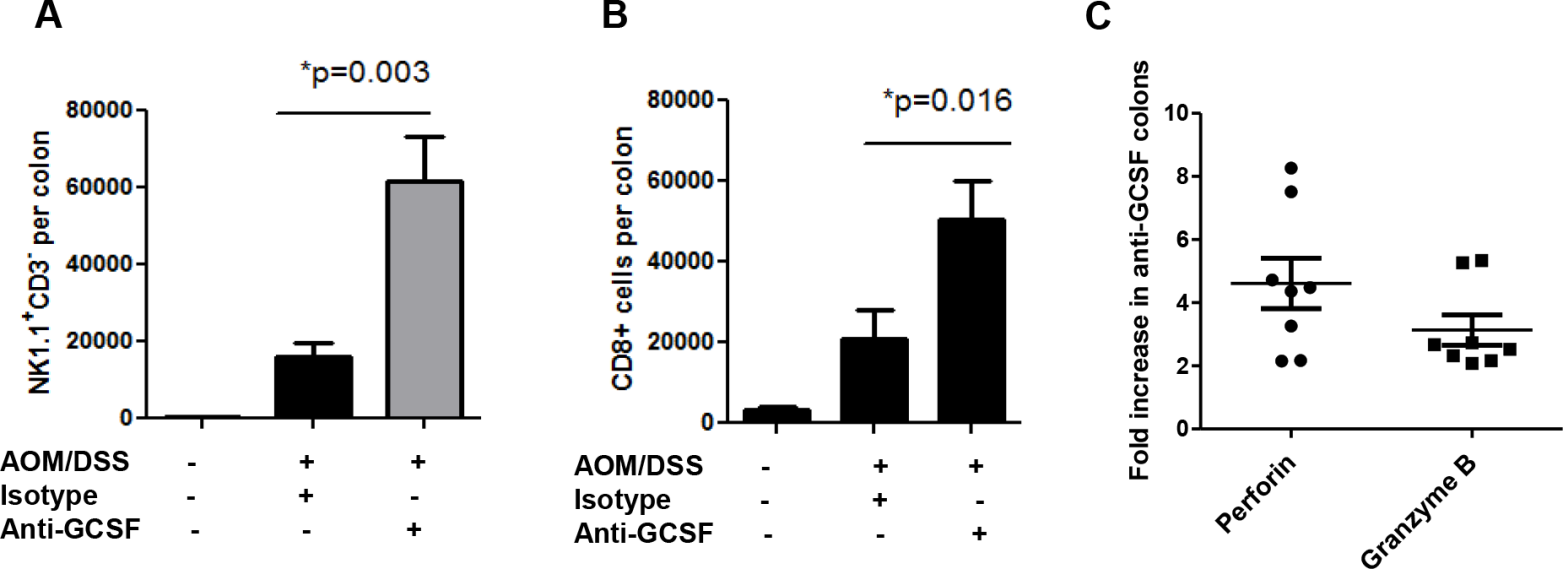
G-CSF neutralizing antibody treatment induces cytotoxic cell influx and responses in AOM/DSS treated mice G-CSF neutralizing antibody induces colon **A.** influx of NK1.1^+^CD3^−^ cells, **B.** influx of CD8^+^ T cells and **C.** increased perforin and granzyme B gene expression. *N* = 7 for sham isotype treated AOM/DSS exposed mice and *N* = 8 for anti-G-CSF AOM/DSS treated mice from duplicate experiments.

Other immune cells that are considered critical for anti-tumor immunity are cytotoxic cells. G-CSF was shown to have potent inhibitory effects on NK cells in graft vs host disease [21], which led us to examine these cells in our mouse groups. With G-CSF blockade, IL-12 was increased in mouse colons (Figure 3D), which is known to be a potent activation factor for NK cell activity. Thus, to examine NK cells, single cell suspensions from colon neoplasms and surrounding tissues were stained for NK1.1 and CD3 to rule out NKT cells. NK1.1^+^CD3^−^ cells were found at dramatically higher numbers (greater than a 3-fold increase) in anti-G-CSF treated mouse colons compared to isotype control treated mouse colons (Figure 4A). In addition to NK cells, CD8^+^ T cells are also potent cytolytic cells. Upon examination of CD8^+^ T cell influx into mouse colons, anti-G-CSF treated mice displayed a greater than a 2-fold increase in number compared to isotype control treated mouse colons (Figure 4B). Furthermore, to investigate the potential activity of cytotoxic cells, gene expression of the cytolytic factors perforin and granzyme B were tested. These factors were increased in the mouse colons of anti-G-CSF treated mice compared to isotype control treated mouse colons (Figure 4C). Perforin was increased by 4.61-fold and granzyme B by 3.14-fold compared to the colons of control mice, thus demonstrating that NK cells and CD8^+^ T cells may be increased in number or exhibit increased activity upon G-CSF depletion. These data indicate a previously unreported observation that blockade of G-CSF may increase the influx and anti-tumor activity of NK and CD8^+^ T cell function in mouse colons.

### Anti-G-CSF treatment changes CD4^+^ and CD8^+^ cell response in mouse colons

**Figure 5:**
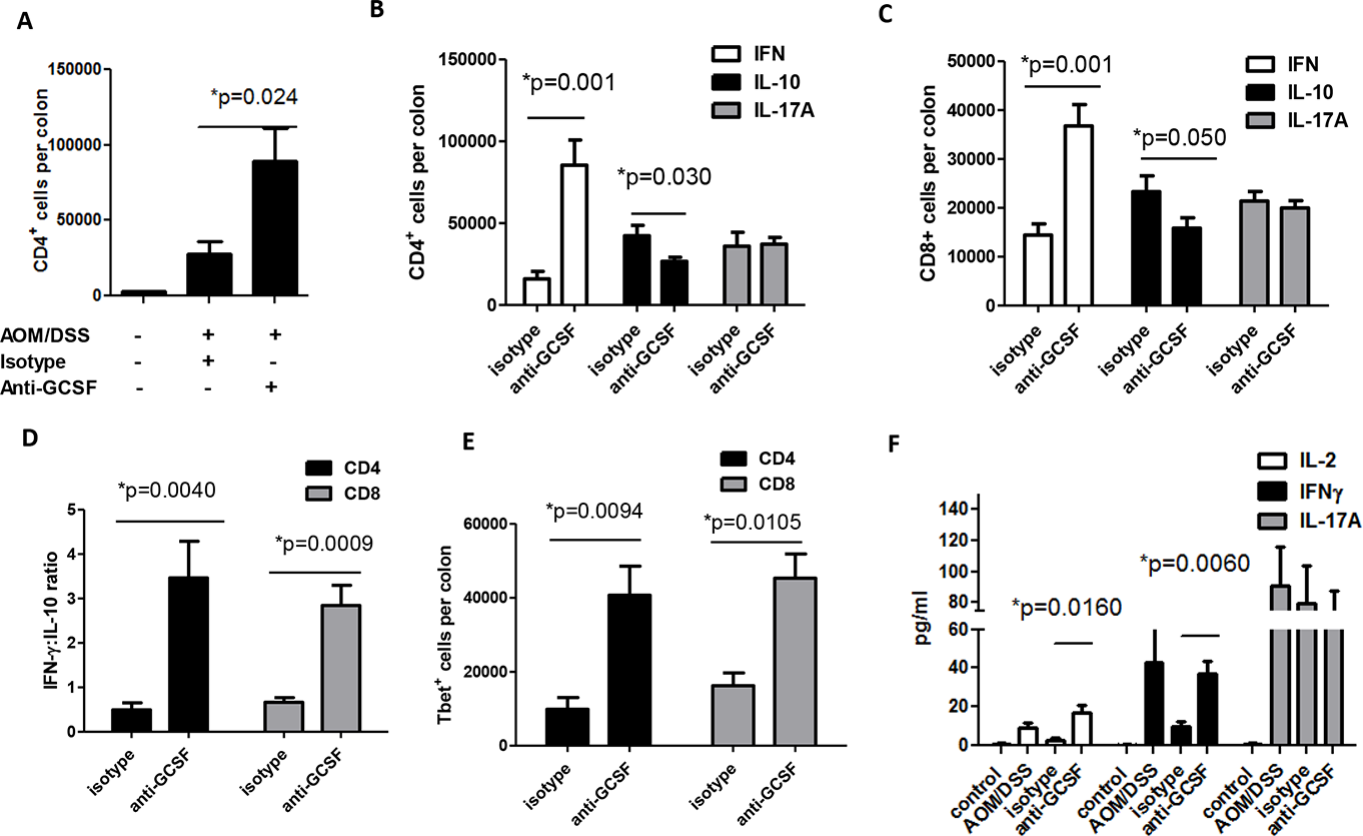
G-CSF neutralizing antibody changes T cell responses in AOM/DSS treated mice Upon anti-G-CSF treatment **A.** colon CD4^+^ were increased and **B.** the number of IFNγ producing CD4^+^ and **C.** CD8^+^ were increased, while IL-10 producing cells were decreased along with a **D.** drastic change in the ratio of IFNγ:IL-10 producing cells. **E.** The number of Tbet expressing CD4^+^ and CD8^+^T cells were also increased in the colons of anti-G-CSF treated mice. **F.** Colon tissue supernatants from anti-G-CSF treated mice were increased in IL-2 and IFNγ production compared to isotype control treated mice and sham PBS injection mice by multiplex array. *N* = 7 for PBS control and isotype treated AOM/DSS exposed mice and *N* = 8 for anti-G-CSF AOM/DSS treated mice from duplicate experiments.

Studies on the effects of G-CSF on T cell responses are limited. However, in diseases such as diabetes and graft vs host disease, G-CSF was shown to support the accumulation of regulatory T cells (Tregs) [11, 13]. These studies, in addition to the high levels of IL-10 in organ culture supernatants (Figure 3D), led us to further examine the T cell responses in isotype vs anti-G-CSF treated mice. Colon neoplasms and surrounding microenvironment tissues of mice administered anti-G-CSF were stained for CD4^+^ T cells. Similar to CD8^+^ T cells in Figure 4B, the number of CD4^+^ were also elevated with a 3-fold increase in number compared to the isotype control group (Figure 5A). To further examine the phenotype of CD4^+^ and CD8^+^ T cells, intracellular cytokine staining was performed for IFNγ as a potential anti-tumor response, IL-10 for a potential Treg/inhibitory phenotype, and IL-17A for an inflammatory phenotype [22]. For both CD4^+^ and CD8^+^ T cells, there was a sizeable increase in IFNγ producing cells, 5-fold for CD4^+^ and 3-fold for CD8^+^ cells (Figure 5B and 5C). A significant decrease was also observed in IL-10 producing cells, but IL-17A expressing cells remained unchanged between anti-G-CSF and isotype control treated groups. For Th2 responses, IL-4 was also examined, but was not found by flow cytometry or in organ culture, which may be typical of the B6 mouse background. Given that the numbers of CD4^+^ and CD8^+^ T cells were increased in anti-G-CSF treated mice, the ratio of IFNγ:IL-10 producing CD4^+^ and CD8^+^ T cells was also examined and found to be markedly higher in the anti-G-CSF treated group compared to the isotype control group (Figure 5D). Since there was a drastic change in IFNγ producing cells, the transcription factor for Th1 cells, Tbet, was examined in CD4^+^ and CD8^+^ T cells and found to be highly expressed in T cells in colons from anti-G-CSF treated mice (Figure 5E). To further support this finding, IL-2, as a general marker for activated T cells, as well as IFNγ, and IL-17A were measured in organ culture supernatants. Total IL-2 and IFNγ production were substantially enhanced in anti-G-CSF treated mouse colon tissues compared to isotype control, while IL-17A was not significantly changed (Figure 5F). Total IL-10, which may be produced by both macrophages and T cells, was also markedly decreased in the colons of anti-G-CSF treated mouse (Figure 3D). Taken together, these data denote that anti-G-CSF treatment induced protective T cell responses through IFNγ and led to increased T cell activation through IL-2 production. Thus, G-CSF may have inhibitory effects on anti-tumor effector T cell activity in the tumor microenvironment, which is a novel finding signifying an important role for this cytokine in inhibition of protective anti-tumor immunity.

## DISCUSSION

G-CSF is a pro-inflammatory cytokine that is well documented to mature and mobilize neutrophils from bone marrow. Despite its known effects on neutrophils, G-CSF has been largely overlooked for a role in tumors and the tumor microenvironment. Our studies indicate that G-CSF may have unrecognized, critical tumor-promoting functions in gastrointestinal cancers. We first examined this in human tumors where we found that G-CSF and G-CSFR expression were highly associated with lymph node metastasis, suggesting a link with poor disease outcome [7]. *In vitro*, we further found that recombinant G-CSF treatments of colon cancer and gastric cancer cells induced proliferation and migration. In addition to our human study, several others have noted that G-CSF expression in solid tumors promotes tumor growth. In particular, in one study of human skin cancer, G-CSF promoted malignant progression of tumor cells [23]. Furthermore, in another study of bladder cancer, G-CSF production by tumor cells was associated with extensive tumor growth and poor clinical outcome [24]. There are also several clinical reports of aggressive tumors in gastric and cervical cancers that produce high levels of G-CSF [5, 25–27]. Based on our findings of high levels of G-CSF and G-CSFR expression in human CRC tumors that was associated with lymph node metastasis [7], we wanted to investigate if blockade of G-CSF would be protective in CRC. We found anti-G-CSF treatments to be very effective at reducing the number and size of neoplasms in the AOM/DSS mouse model of CRC. Since this model is a highly reproducible colitis-associated model that mimics IBD-associated cancer [28], G-CSF may promote both colitis-associated and sporadic colorectal cancers.

Given the well-known role of G-CSF in mobilizing bone marrow neutrophils, most of the attention in G-CSF research has been focused on this function. Thus, we predicted that G-CSF may affect the immune response in the AOM/DSS model. No change in neutrophil influx was detected so it is possible that neutrophils are recruited before anti-G-CSF treatment or by other chemokines that are induced by DSS treatment such as MIP-2, KC, and CXCL2 [29–31]. Macrophages are thought to be major tumor promoting cells in some cancers, particularly in CRC [32, 33] and macrophages expressing both M1 and M2 markers were recently described in the AOM/DSS model [34]. In this study, we examined the impact of G-CSF neutralization on macrophages in mouse colons. Similar to neutrophils, the number of cells was not changed; however, the production of IL-10 was decreased while IL-12 was increased. These findings represent a change in phenotype from what is typically understood to be pro-tumorigenic to anti-tumorigenic [35]. Although novel, this finding is supported by another study where G-CSF has been shown to have immunoregulatory effects when exogenously administered to human patients or animals [30]. These studies also reported that G-CSF inhibits LPS-induced IL-12 production from bone-marrow derived dendritic cells *in vitro* and suggested that G-CSF may induce IL-10 production in cells of the monocyte lineage. In the AOM/DSS model, macrophages expressing markers of both M1 and M2 phenotypes were recently shown to be present [34], but G-CSF blockade may change the balance of this phenotype toward M1.

Given that IL-12 also promotes NK function and protective immunity in CRC [36], we further examined NK cells in mouse colons after anti-G-CSF treatments. We found a substantial increase in NK cell numbers, similar to other studies indicating that IL-12 induced NK cell expansion [37, 38]. IL-12 has been shown to increase cytotoxic perforin expression by NK cells [39], and we found increased perforin along with granzyme B. In further support of a direct role for G-CSF on NK cell function, in graft vs. host disease G-CSF was shown to be a potent inhibitor of NK cell function [21], and transplantation patients who received G-CSF therapy showed impaired NK cell function and diminished NK cell numbers in peripheral blood [40]. These studies support an inhibitory role for G-CSF on NK cells.

Finally, we examined CD4^+^ and CD8^+^ T cells and found increased numbers in the colons of mice that had been treated with anti-G-CSF compared to isotype control. Both CD4^+^ and CD8^+^ T cells were increased in number, IFNγ producing cells were increased, and IL-10 producing cells were decreased concurrently. These results represent a shift from a regulatory phenotype to an anti-tumorigenic phenotype and suggest that G-CSF plays a role in promoting IL-10 producing immune cells that are likely inhibitory in the tumor microenvironment. This concept is supported by the work of others in diseases where inhibitory immune responses are helpful, such as graft vs host disease and diabetes. In several studies, G-CSF induced IL-10 production by T cells [11, 12, 41]. Additionally, there are two studies suggesting that G-CSF may induce IL-10 production by antigen presenting cells [42, 43] also suggesting a more general role for G-CSF in IL-10 production.

Our findings provide evidence that anti-G-CSF treatment induces protective anti-tumor immunity in the colons of AOM/DSS treated mice. The decrease in IL-10 with concurrent increase in IL-12 producing macrophages in mouse colons with anti-G-CSF treatment suggests a shift from a pro-tumorigenic to an anti-tumorigenic phenotype. NK and CD8^+^ cells numbers were heightened along with the presence of perforin and granzyme B gene expression suggesting enhanced cytotoxic function. Furthermore, an increase in potentially anti-tumorigenic IFNγ producing CD4^+^ and CD8^+^ T cells also indicate improved anti-tumor immunity. Taken together, these results reveal that anti-G-CSF treatment has potent immune-modulating effects that demonstrate the potential for anti-G-CSF as a therapeutic approach for human cancers expressing this cytokine.

## MATERIALS AND METHODS

### Animal experiments

Female C57Bl/6 (B6) mice from Harlan Laboratories (Houston, TX) were housed under pathogen free conditions. Under approval of the UNM IACUC, at 6 weeks of age the mice received one 12.5mg/kg intraperitoneal (IP) injection of AOM. During days 5–10 and 26–31 after injection mice received 2.5% DSS in drinking water and on days 47–51 they received 2% DSS. At day 54, mice were administered either 25 μg of IgG1 or anti-G-CSF antibody (R&D Systems MAB005 and MAB414) via IP injection 3 times a week for 3 weeks and sacrificed on Day 80 using CO_2_. Neoplasms were counted and measured L x W (mm) under a dissecting microscope. Colon tissues were divided for flow cytometry, quantitative real time PCR (qRT-PCR) and histological examination.

### Quantitative real-time PCR

RNA was isolated using Ribozol (Amresco, Solon, OH) according to the manufacturer's instructions. RNA concentrations were measured using a Nanodrop (Thermo Scientific, Wilmington, DE, USA). qRT-PCR was performed according to Applied Biosystems' (Foster City, CA, USA) two-step protocol as we have previously published [7].

The RT reaction mixture includes random 2.5 μM hexamers, 500 μM dNTPs, 0.4 U/μL of the RNase inhibitors, 5.5 mM MgCl_2_, MultiScribe Reverse Transcriptase (3.125 U/μL) and its buffer, and 1 μg of cellular RNA. The RT step was performed according to the following protocol: 10 min at 25°C, 60 min at 37°C, 5 min at 95°C. Obtained cDNA samples were stored at −80°C and used for the PCR reaction step. The PCR reaction mix was prepared using the Assays-on-Demand™ gene expression assay mix (Applied Biosystems) for mouse 18S, G-CSF, G-CSFR, perforin, and granzyme B (a 20X mix of unlabeled PCR primers and TaqMan^®^ MGB probe, FAM dye-labeled) and 2 μL of cDNA were added to the PCR reaction mix. The reaction was carried out according to the following protocol: 2 min at 50°C, 10 min at 95°C (1 cycle), and 15 sec at 95°C and one min at 60°C (45 cycles) on Applied Biosystem's StepOnePlus instrument. The endpoint used in real-time PCR quantification, CT, was defined as the PCR cycle number that crossed the signal threshold. Quantification of cytokine gene expression was performed using the comparative CT method (Sequence Detector User Bulletin 2; Applied Biosystems) and reported as the fold difference relative to the human housekeeping gene, 18S mRNA.

### Flow cytometry

Mouse colons and lymph nodes were processed for single cell suspension as previously published [44] and multi-color staining performed according to standard FACS staining protocols. Briefly, tissues were treated with collagenase (I, II, and IV, Sigma Aldrich, St. Louis, MO) and dispersed twice using the gentleMACs tissue dissociator (Miltenyi Biotech, Cologne, Germany). Cell suspensions were incubated in complete RPMI media for 24 hours before staining for flow cytometry. Multi-color staining was performed according to standard surface and intracellular FACS staining Biolegend protocols (Biolegend, San Diego, CA). Antibodies used in this study were anti-Ly6G-APC (clone1A8, Biolegend), anti-F4/80-PCP (PerCP/Cy5.5), (Biolegend, BM8), anti-IL-12p35-APC (eBioscience, 4010p35), anti-IL-10-FITC (Biolegend, JES5-16E3), anti-NK1.1-PCP (eBioscience, PK136), anti-CD3-FITC (Biolegend, 145-2C11), anti-CD4-PCP (Biolegend, GK1.5), anti-CD8-PCP (Biolegend, 53-6.7), anti-Tbet-FITC (Biolegend, 4B10), anti-IFNγ-PE (Biolegend, XMG1.2), anti-IL-17A-APC (Biolegend, TC11-18H10.1), and anti-IL-4-FITC (eBioscience, 11-7042-82). All samples were analyzed on a Guava easyCyte 8HT flow cytometer (EMD Millipore, Bellerica, MA, USA), and analyzed using FCS Express software (DeNovo Software, Los Angeles, CA, USA).

### Luminex arrays

Colon tissue pieces were cut to 8mg and put into complete RPMI for 12 hours. Supernatants were collected and cytokines were measured by multiplex bead array (EMD Millipore, Bellerica, MA) using a Luminex™200 machine.

### Statistical analysis

Results were expressed as the mean ± SE of data. Differences between means were evaluated by ANOVA using Student's *t*-test for multiple comparisons. Values of *P* < 0.05 were considered statistically significant.
